# Cambra system in patients awaiting hematopoietic progenitor cell transplant and high caries risk

**DOI:** 10.4317/jced.53617

**Published:** 2017-05-01

**Authors:** Ana Hernández-Fernández, Antonio-José Ortiz-Ruiz, Felipe De Arriba-de la Fuente, Vicente Vicente-García, Pastora Iniesta-López-Matencio, Ricardo-Elias Oñate-Sánchez

**Affiliations:** 1 DDS. Associate Lecturer. Faculty of Dentistry, University of Murcia; 2 MD, PhD, DDS. Senior Lecturer. Faculty of Dentistry, University of Murcia; 3 MD, PhD. Hematology and Oncology Services, Morales Meseguer Hospital

## Abstract

**Background:**

Recent times have witnessed a significant increase in the number of patients affected by problems related to oncological treatment Aims of this study is to evaluate dental affectation among patients awaiting hematopoietic progenitor cell transplant (HPCT), and they showed high caries risk, so it should establish a protocol prior to transplantation.

**Material and Methods:**

The study included 72 patients due for HPCT. Clinical and radiological explorations were performed and oral photos taken. The amount of caries, missing teeth and fillings were registered for each patient. CAO, DMFS and Restoration Indices were calculated.

**Results:**

83% of patients presented caries. 48 patients (67%) had lost at least one tooth. Only 32 patients (44%) had received some sort of conservative treatment. The average CAO index value obtained was 10.37. The DMFS index showed an average of 27.06 affected surfaces. Of the 72 patients studied, 40 (56%) showed a restoration index value of zero.

**Conclusions:**

These patients presented a high number of carious teeth and a low restoration index. The presence of so many possible septic foci in an individual, who will later become susceptible to infection, highlights the importance of preventative treatment and bucco-dental restoration within this patient population. These patients with a high caries risk can be treated with CAMBRA system.

** Key words:**Hematopoietic progenitor cell transplantation, high caries risk, state of oral health, haematological disease, CAMBRA system.

## Introduction

Recent times have witnessed a significant increase in the number of patients affected by problems related to oncological treatment (1,2). This increase has been due to the major advances in recent years in both the treatment of different types of cancer and in increased knowledge of their etiopathogenesis, so that 50% of patients diagnosed with a neoplastic process are now treated successfully. Even so, patients perceive the diagnosis of cancer as one of the most traumatic and perturbing events of their lives.

Among neoplasias, haematologic neoplasias are a group with high prevalence, leukemias and lymphomas being the most frequent (3).

Treatment of hematologic neoplasias includes chemotherapy (CT), radiotherapy (RT) or a combination of the two. Many of these patients will also undergo hematopoietic progenitor cell transplant (HPCT), not only for the eradication of the disease but also to palliate the collateral effects of treatment that involves high doses of cytostatics or radiation (4).

It is also known that one of the principal causes of death among cancer patients is infection (5,6). The oral cavity is a common site of infectious processes and other pathologies which can affect cancer patients (7). Often in cancer treatment, the mouth is relegated in planning therapeutic procedures aimed at correcting the neoplastic process and if oral complications appear they can interrupt therapy. Oral complications associated with cancer treatment can produce localized discomfort, severe pain, nutritional deficiencies, as well as delaying the patient’s recovery, with the resulting increase in health care costs caused by longer hospital stays, and can even endanger the patient’s life.

Most researchers (5-12) agree that ensuring a number of conditions of bucco-dental health, prior to oncological treatment, will minimize its negative effects. Careful oral examination must be made before the initiation of the antineoplastic therapy. This will identify and eliminate potential sources of infection, decrease the incidence of mucositis and reduce hospital charges.

Cancer prevention, cure, and management is a major undertaking, and in this context the specific needs of children and adolescents with cancer are often ignored, probably due to the fact that children represent less than 2% of all cancer sufferers. Even so, neoplasias among children are very different from those that appear in adults, both in type, and because of the effects that anti-cancer therapies have on children’s or adolescents’ growth. For this reason, specific strategies for management and assessment should be included in approaches to treating the different types of childhood cancer, and this includes the application of high quality, effective, and safe protocols (13).

On this basis, in order to determine dental affectation among a population awaiting HPCT, this study examined the bucco-dental health and corresponding treatment needs of these patients, who were candidates for HPCT as treatment for neoplastic processes.

Initiating an oral care strategy using CAMBRA (Caries Management by Risk Assessment) (14) system can reduce oral problems associated with chemotherapy.

Assessing the risk of caries is the first step towards its management. The CAMBRA system is based on a series of formulae, which help determine the risk of caries in relation to the patient’s age, and evaluate the balance between signs of disease, risk factors, and protective factors.

The American Academy of Pediatric Dentistry (AAPD) recognizes that caries assessment and management systems can help dentists and cancer specialists in decision-making and treatment planning. These will be based on the risk of caries, the patient’s situation, and level of commitment, and constitute essential elements in contemporary clinical care of infants, children, and adolescents, but it can also be used in adults. The CAMBRA system aims to educate dental professionals, as well as practitioners in other branches of health-care, in caries risk assessment in dentistry, as well as facilitating clinical decision-making related to caries diagnosis, fluoride application, dietary advice, and restoration protocols. Caries risk assessment models involve a combination of factors including diet, exposure to fluoride, host susceptibility, and oral microflora; these factors also interact with a range of socio-cultural and behavioral variables.

## Material and Methods

The study included 72 patients who were due to receive hematopoietic progenitor cell transplantation. They were referred to the Special Patients Teaching Unit at the University Dental Clinic by the Oncohematology Service at Morales Meseguer Hospital.

Oral exploration was performed at least fifteen days before patients were to be hospitalized at the Transplant Unit.

Study inclusion criteria were: 1. Patients who had been diagnosed with either cancers of different types and at different locations or myelodysplastic syndrome. 2. Treatment of these pathologies was to include HPCT. 3. Patients with teeth, so that the state of oral health could be assessed; completely edentulous patients were excluded.

Having obtained informed consent from each patient, clinical and radiological explorations were performed and oral photos were taken.

The materials used for clinical exploration were: patient clinical history, an exploration kit and other related disposable materials. Patient clinical histories were specially designed by the Special Patients Units to meet the requirements of the present study. The exploration kit consisted of: two dental mirrors Nº 5 (Actual®) with stainless steel handles (Roeko®), dental tweezers 2885 (Martin®), a 17-22 (Hu-Friedy®) exploratory probe and a PCP 11 5B (Hu-Friedy®) periodontal exploratory probe.

Radiographic exploration consisted of orthopantomograms (OPG). The orthopantomography equipment used was the PM 2002 CC (Plamenca®). The radiographic plate was the Kodak® La T-Mat G/RA. In some cases it was necessary to make intraoral radiographs using Kodak® film, Ultra Speed DF-58.

Radiographic images necessarily included all the details to be analyzed for the purposes of the study with sufficient image quality for clear visualization of these features and structures.

The photo camera used was a Yashica Reflex model FX-3. Intraoral mirrors of various sizes and shapes were used for taking the photos, as well as lip separators for children and adults.

Data collection was performed by a single practitioner and was recorded by a second member of the team who acted as auxiliary. Both the examiner and the data compiler were fully trained and all evaluations were calibrated according to WHO criteria by means of the Kappa coefficient of determination (coefficient= 0.73) 

The patient was received at the dental clinic and personal details were recorded before taking the patient for orthopantomography (OPG). An odontogram was filled out which included gingival data. Oral photos were then taken. When the session had come to an end the indices were calculated.

Diagnosis of caries followed World Health Organization (WHO) criteria (16). Any case in which it was not clear whether or not these criteria had been met was regarded as caries-free. As well as registering the extent of caries, number of missing teeth and number of fillings per patient, the following indices were calculated: Caries and Obturations Index (CAO) DMFS (Decayed, Missing, and Filled Surfaces) and Restoration Indices.

All the data compiled was subjected to statistical analysis using SPSS software. Variables were analyzed both descriptively and analytically. For the descriptive study, qualitative variables were calculated with frequency distribution, whilst for quantitative variables, means were calculated as a centralization measure; standard error of the mean and standard deviation were used as measures of dispersion; and maximums and minimums were used to establish ranges. Relations between qualitative and quantitative variables were performed using the student t-test whenever two averages were involved; when more than two were involved, the analysis of variance test (ANOVA) was applied. For relationships between qualitative variables, contingency tables were used and Pearson’s chi-squared test was applied; analysis of residues was also performed when necessary. In all cases, a difference between groups or a relation between variables was taken as statistically significant when the significance level was less than 0.05 (*p*<0.05).

## Results

Of the 72 subjects, 42 were men and 30 were women. The average age of subjects was 42.2 ± 1.9 years, the youngest patient being 12 years old and the oldest 69.

All patients were due to undergo HPCT and had been diagnosed with different neoplastic processes, although most of these were blood neoplasias. The patients were subjected to autologous (42 patients) or allogeneic transplant (30 patients).

**Figure 1:**
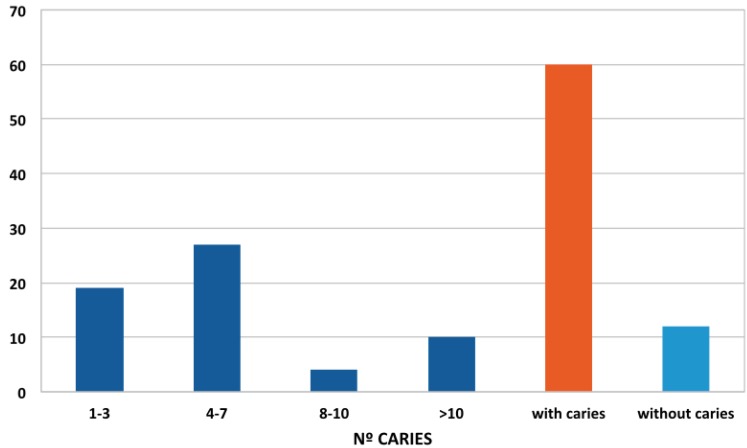
Distribution of number of caries according to degrees of severity.

With regard to carious pathology, 83% (60 patients) suffered caries (Fig. 1). Furthermore, 14 % had more than ten caries lesions (Fig. 1). In patients with caries, men predominated (35 men, 25 women). The overall average caries, regardless of gender, was 4.69, with standard error of the mean of 0.49 ( Table 1). When the number of caries was related to age ranges, the age interval with the highest average number of caries was the 45-54 age group ( Table 2).

**Table 1:**
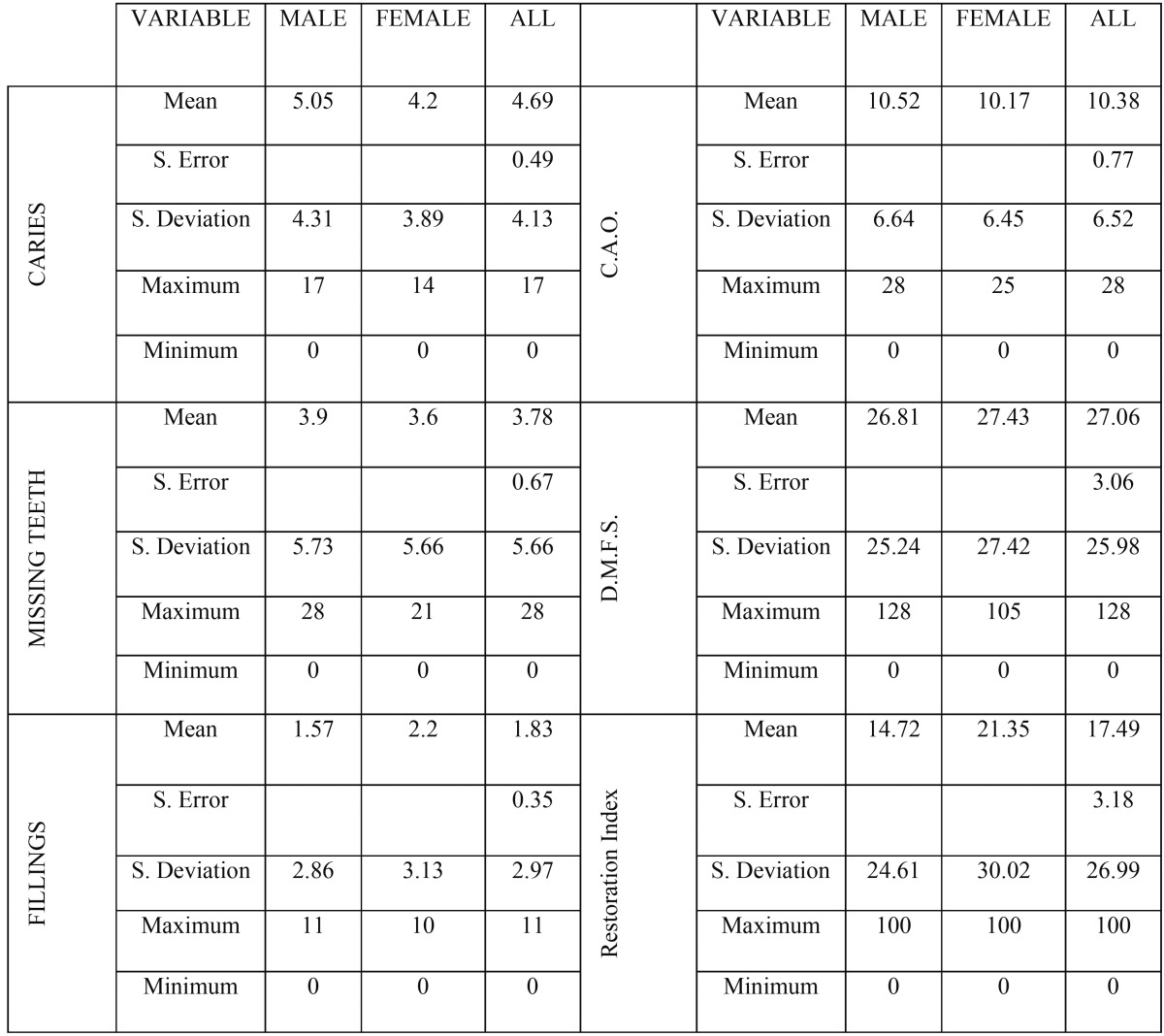
Distribution and dispersion of caries, missing teeth, fillings, CAO and DMFS Indices and Restoration Index.

**Table 2:**
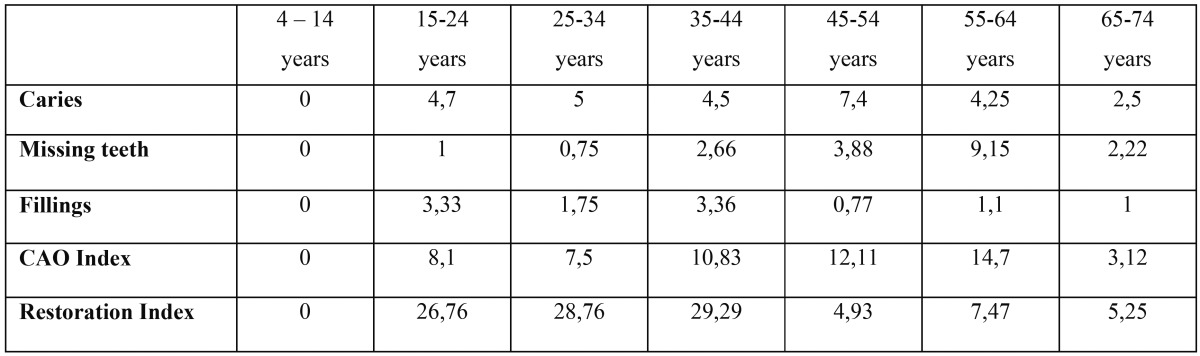
Distribution of caries, missing teeth, fillings, CAO Index and Restoration Index by age intervals.

**Figure 2:**
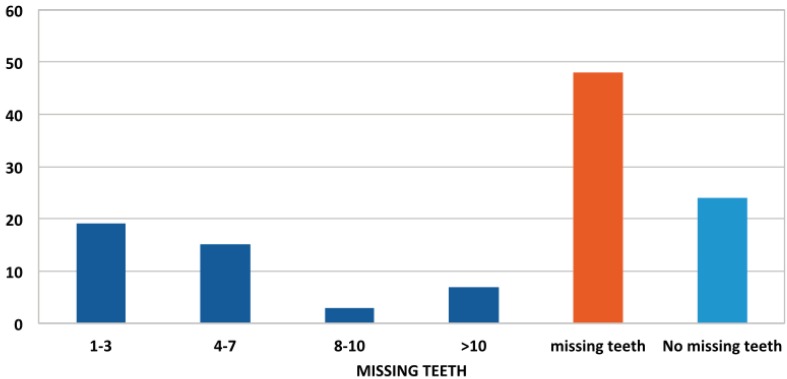
Distribution of the number of missing teeth by degrees of severity.

Twenty-four patients had all their teeth (33%), while 48 had lost at least one tooth (67%) (Fig. 2). Of the 48 with missing teeth, 14.5% (7 patients) had more than ten absent teeth (Fig. 2). When distribution by gender was studied, men predominated. The average number of teeth lost per patient was 3.78, with standard error of the mean of 0.67 ( Table 1). Analyzing the numbers of absent teeth in relation to age groups, as age increased, so did the number of missing teeth ( Table 2).

**Figure 3:**
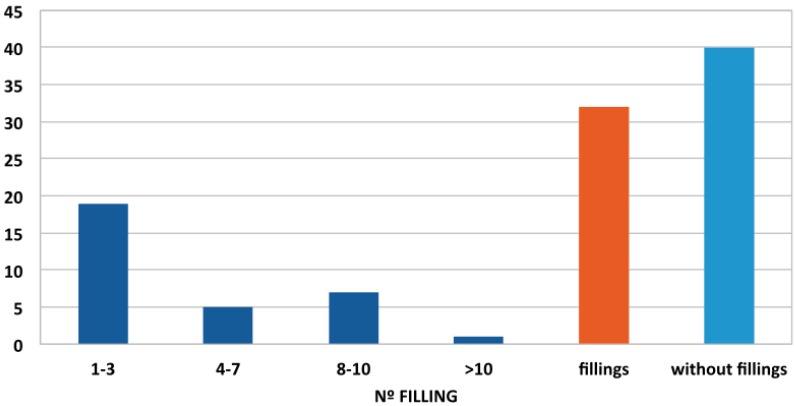
Distribution of number of fillings by degrees of severity.

Regarding the number of fillings among the study population, there were 40 patients who had not received any type of conservative treatment (56%), while 32 had received at least one filling (44%) (Fig. 3). Of the 40 patients who had not undergone any restorative treatment, 36 (90%) had caries. When the distribution of fillings was studied by gender, men predominated among the patient group who had not received any restorative treatment. The average number of fillings per patient, regardless of age or sex, was 1.83, with a standard error of the mean of 0.35 ( Table 1). Distribution by age showed that younger patients had received higher numbers of restorations ( Table 2).

Only three patients obtained a 0 value in the CAO index, with 69 obtaining values greater than 0 which represents 95.8% of the sample. The average CAO index value was 10.37, with standard error of the mean 0.77 ( Table 1). Analyzing CAO index values in relation to age intervals, the highest values fall within the 55-64 year interval ( Table 2).

The DMFS index, like the CAO index, obtained only three patients with the 0 value, in other words, who had no surfaces affected by caries. The majority of patients were within an interval of between four and fourteen affected surfaces. The average was 27.06 affected surfaces, with standard error of the mean of 3.06 ( Table 1).

The restoration index reflects the level of motivation and importance that bucco-dental health has for the individual patient. The ideal would be 100%, but COA index values are determined by the sample’s obturation component.

Of the total of 72 patients, 40 (56%) showed a restoration index value of 0. This indicates that these 40 individuals had undergone no restorations. Only three patients (4%) showed a 100% restoration index, in other words, all teeth had been restored and these patients showed no incidence of caries and had all their teeth. The mean restoration index was 17.49, with a standard error of the mean of 3.18. Distribution by gender reflected a higher restoration index among women than men ( Table 1). Analyzing the restoration index by age, it was higher among younger patients ( Table 2).

## Discussion

Cancer is one of the major health problems in the world. According to the World Health Organization (WHO) (1), about eleven million people all over the world are diagnosed with malignant tumor and 6.7 million die from cancer every year. Of these 6.7 million, 56% are men and 44% women. There are currently 22 million individuals in receipt of medical attention as a result of neoplasia.

In Spain, one in three men and one in five women will develop some type of cancer before the age of 74, which indicates the high frequency of the pathology (17). Clearly, it is important to research and analyze all possible problem that these patients can present, in all the aspects of their lives: this is the first step towards resolving them.

The patient sample studied is representative both in gender distribution, age and tumoral pathology of other samples described in the literature. Regarding gender, in the present population, 58% were men and 42% women. This is in accord with patient groups in other research (3,18), with a slight male predominance, but without statistically significant difference between the sexes. The average age was 42.4 years, which corresponds to the fact that most of these malign processes appear in adulthood; most authors give mean ages slightly above the present sample (19,20). However, the most numerous age group was between 55 and 64, representing 27.78% of the total number of patients. This is in closer agreement with contemporary research. Furthermore, it should be remembered that patients aged over the 55-65 interval are typically unsuited to HPCT (20-23). This may explain why the mean age in this patient population is younger, despite the fact thathematologic neoplasias notmally appear at an older age. The fact that some patients were diagnosed with lymphoid leukemia could also be a factor that would bring down the mean patient age. Like gender and age, tumoral pathology is also concurrent with data reviewed in the literature (3).

In Spain, there has not been much research that assesses the dental state of patients awaiting cancer treatment and even less involving HPCT, and so the results of the present study have been compared to bucco-dental health surveys of the general population in Spain (24).

When the number of caries was analyzed, 83% of patients presented active caries, regardless of age or gender. Comparing this finding with data published in the most recent (2010) Bucco-Dental Health Survey carried out among the general public in Spain (24), the percentage of patients with caries in the present study (83.3% in the patient group aged between 35 and 44 years) is higher than the percentage of caries in any age group in the survey (which found 56.5% (IC% 48,3-59,9) for the same age group).

With regard to the number of missing teeth among the present patient sample, this variable was clearly influenced by age, with a statistically significant association (ANOVA *p*<0.0001), so that as age increased the greater were the numbers of missing teeth. This corresponds to the general population according to the bucco-dental health survey consulted (24).

The average numbers of restored teeth in the Spanish population was greater (4.39) than the mean treated among the present patient sample (3.66), perhaps because these patients prioritize their disease treatment, leaving oral health relegated to the background.

The CAO is an index that has to be analyzed in relation to age. In the present study values for this variable rose with each age interval (ANOVA *p*<0.001) in the same way as the 2010 bucco-dental health survey. The DMFS Index was also seen to be influenced by patient age (ANOVA *p*< 0.0001).

The fact that both the CAO and DMFS indices and numbers of missing teeth are influenced by age, while the number of caries and fillings are not, makes it clear that the increased indices are due to the numbers of teeth lost. This also occurred among older age groups in the Spanish national bucco-dental health survey.

The mean determined for the restoration index in the present patient sample was 17.49%, being higher for women than for men, and for younger patients. This value is much lower than values registered by Llodra Calvo JC *et al.* (authors of the 2010 national bucco-dental health survey) (24) for all ages (54%), with the exception of the 65-74 age interval.

The restoration index was seen to be influenced by age in an inverse relation, so that the older the patient, the lower the restoration index. This also concurs with the Bucco-Dental Health Survey (24), when the highest and lowest values are compared.

In conclusion, when all data are analyzed, they give a general impression that patients due for HPCT present a higher number of carious teeth, higher than would be expected among the general population and a low restoration index. Furthermore, their state of oral health was seen to worsen with age. It might be believed that the high incidence of caries and missing teeth is a consequence of earlier chemo- and radiotherapy. However, the time passed between the CT and RT applications and HPCT is usually short and not enough time to justify attributing the patients’ state of oral health to the cancer treatment received. This is likely to be the result of poor oral hygiene in the long-term.

The presence of so many potential septic foci in a patient who will be susceptible to infection as a result of the treatment he/she is about to undergo, means that there is a need for these patients to receive bucco-dental examination and treatment prior to HPCT. This concept should be made known among health professionals in the field of hematology-oncology in particular and health care workers in general.

Oral complications associated with cancer treatment can produce local discomfort, intense pain, deficient nutrition, slow the patient’s recovery, with increased health-care costs due to longer hospital stays. They can even endanger the patient’s life. Initiating an oral care strategy using CAMBRA system can reduce oral problems associated with chemotherapy.

Although this protocol is used mainly in children and adolescents, its use can be beneficial in adult patients with a tendency to have a high risk of tooth decay due to their illness. Preventive measures, treatment guidelines and the establishment of a comprehensive monitoring protocol proposed by the CAMBRA system are totally appropriate for patients with hematologic malignancies.

Tracking a standardized protocols for adult patients in general and cancer patients in particular ensures the success of treatment and their long-term stability. This is because of all individuals normalizes alike, studying their risk factors. Besides all the preventive and therapeutic actions they are well studied and have been proven effective. Likewise, the timing of revisions allows the prevention of relapse.

Introducing CAMBRA system use as a standard practice could also reduce the legal risks involved in dental treatment of these patients. Further initiatives are needed to properly establish these approaches to treatment.
